# Artificial Intelligence in the Diagnosis of Upper Gastrointestinal Diseases

**DOI:** 10.1097/MCG.0000000000001629

**Published:** 2021-11-04

**Authors:** Pierfrancesco Visaggi, Nicola de Bortoli, Brigida Barberio, Vincenzo Savarino, Roberto Oleas, Emma M. Rosi, Santino Marchi, Mentore Ribolsi, Edoardo Savarino

**Affiliations:** * Gastroenterology Unit, Department of Translational Research and New Technologies in Medicine and Surgery, University of Pisa, Pisa; † Department of Surgery, Oncology, and Gastroenterology, Division of Gastroenterology, University of Padua, Padua; ‡ Gastroenterology Unit, Department of Internal Medicine, University of Genoa, Genoa; ∥ Department of Digestive Diseases, Campus Bio Medico University of Rome, Roma, Italy; § Ecuadorean Institute of Digestive Diseases, Guayaquil, Ecuador

**Keywords:** artificial intelligence, gastrointestinal endoscopy, upper gastrointestinal cancer, GERD, *Helicobacter pylori* infection

## Abstract

Artificial intelligence (AI) has enormous potential to support clinical routine workflows and therefore is gaining increasing popularity among medical professionals. In the field of gastroenterology, investigations on AI and computer-aided diagnosis (CAD) systems have mainly focused on the lower gastrointestinal (GI) tract. However, numerous CAD tools have been tested also in upper GI disorders showing encouraging results. The main application of AI in the upper GI tract is endoscopy; however, the need to analyze increasing loads of numerical and categorical data in short times has pushed researchers to investigate applications of AI systems in other upper GI settings, including gastroesophageal reflux disease, eosinophilic esophagitis, and motility disorders. AI and CAD systems will be increasingly incorporated into daily clinical practice in the coming years, thus at least basic notions will be soon required among physicians. For noninsiders, the working principles and potential of AI may be as fascinating as obscure. Accordingly, we reviewed systematic reviews, meta-analyses, randomized controlled trials, and original research articles regarding the performance of AI in the diagnosis of both malignant and benign esophageal and gastric diseases, also discussing essential characteristics of AI.

## WHAT IS ARTIFICIAL INTELLIGENCE (AI) AND HOW IT WORKS

The term AI generically refers to complex computer algorithms that mimic human cognitive functions, including learning and problem-solving.^1^ Machine learning (ML) is a field of AI that involves computer-based methods to elaborate data through algorithms. Conventional ML is based on hand-crafted algorithms in which researchers, based on clinical knowledge, manually indicate features of interest of an input dataset to train the system to recognize discriminative features, and provides appropriate outputs (ie, solving the problem of interpreting given data).^1^ The aim of ML is to find a generalizable model applicable to new data, which were not included in the training dataset, so that the computer can learn to interpret previously unknown information and provides reliable outputs.^2^ Learning techniques are divided into supervised, unsupervised, and reinforcement methods. A supervised learning model learns from known patterns,^2^ and requires the training dataset to contain input-output pairs to map new input to output.^3^ Unsupervised models are designed to classify subgroups of data according to commonalities without an a priori knowledge of groups significance.^3^ In reinforcement learning, the computer learns from its previous errors, adjusting the output over time.^2^

**FIGURE 1:**
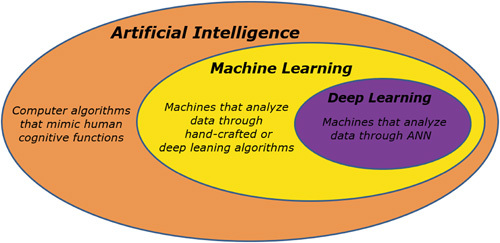
Relationship between artificial intelligence, machine learning, and deep learning. ANN indicates artificial neural network.

**FIGURE 2:**
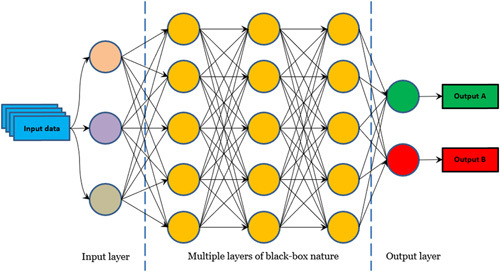
Structure of convolutional neural networks.

Recently, a derivative of ML referred to as deep learning (DL) has enthusiastically broken into the scene (Fig. 1). In contrast to ML, DL is more powerful as it autonomously extracts discriminative attributes of input data through an artificial neural network (ANN), often organized as convolutional neural networks (CNNs), which are constituted of multiple layers of nonlinear functions (Fig. 2).^1^,^4^ AI, ML, and DL are increasingly being integrated into computer-aided diagnosis (CAD) systems that can be applied to gastrointestinal (GI) diseases to improve recognition and characterization of pathology. The main application of AI in the upper GI tract is endoscopy. The ability to recognize endoscopic images depends on individual expertise, being interobserver and intraobserver variability a limit of endoscopic procedures. CAD tools have the potential to successfully assist both trainee and expert physicians to reduce variability in the detection of upper GI pathology, thus increasing the diagnostic accuracy regardless of individual expertise, and virtually overcoming current limitations of esophagogastroduodenoscopies (EGDS).^5^ Besides luminal imaging, AI has been applied to numerical and categorical data describing upper GI pathology to automate and optimize the assessment of diseases, including gastroesophageal reflux disease (GERD), eosinophilic esophagitis (EoE), and primary esophageal motility disorders.

As promising as it is, DL has its own limitations as models cannot apply reason throughout the decision process, and this may be counterproductive.^5^ DL models are black boxes in which the input data and the output (diagnosis) are known, but the processes by which the diagnosis is achieved are not, thus it is difficult to investigate the rationale for the diagnosis made by DL. In this regard, science not only is required to provide answers but also to explain why those are the answers for academic, legal, and ethical implications.^6^ Research is already heading to understand how DL models make decisions to solve interpretability gaps, and methods to understand the process of CNN-based choices are being developed. For example, an option is the use of “heat maps” that indicate what parts of an image the CNN has analyzed or altering model input data to appreciate how the outputs change.^7^ Of note, these highly sophisticated computational systems are not cost-effective at present and could lead physicians to rely on machines more than their clinical judgment while still retaining responsibility for decisions nonetheless.^4^ Despite some limitations, AI ex vivo and in vivo real-time support in decision-making is a fascinating hot topic in our time. Accordingly, we reviewed current knowledge regarding the application and performance of AI in the diagnosis of several esophageal and gastric diseases.

## METHODS

We searched MEDLINE (PubMed), EMBASE, EMBASE Classic, and the Cochrane Library (from inception to April 2021) to identify systematic reviews, meta-analyses, randomized controlled trials, and original research articles reporting the performance of AI systems in the instrumental or clinical diagnosis of several esophageal and gastric diseases. The following terms were searched: AI or machine learning, deep learning. We combined these using the set operator AND with studies identified with the following terms: *esophageal and gastroduodenal endoscopy*, *cancer, carcinoma*, *neoplasia*, *Barrett’s esophagus*, *esophagitis*, *gastro-esophageal reflux disease*, *GERD*, *eosinophilic esophagitis*, *EoE*, *motility disorder*, *varices*, *gastric cancer*, *atrophic gastritis*, *Helicobacter pylori infection.* All terms were used as MeSH terms. Restriction to English language was applied. We screened titles and abstracts of all citations identified by our search for potential suitability and retrieved those that appeared relevant to examine them in more detail. The “snowball strategy” (ie, a manual search of the references listed in online databases publications) was performed to increase sources of information.

### AI in Barrett’s Esophagus (BE) and Early Esophageal Adenocarcinoma (EAC)

The replacement of squamous esophageal epithelium with intestinal metaplasia containing goblet cells defines BE, which represents a well-known preneoplastic lesion for the development of EAC. BE has a predictable course through intermediate stages, namely BE with low-grade dysplasia, high-grade dysplasia (HGD), intramucosal carcinoma, and eventually invasive EAC.^8^ Thus, BE-derived EAC is preventable and amenable to surveillance strategies.

In 2018, esophageal cancer (EC) was estimated to account for 508,000 deaths, being the seventh most common cancer and the sixth cause of cancer death worldwide. Histologically, EAC accounts for 20% of all ECs, and its main risk factors include GERD and high body mass index.^9^ When diagnosed in advanced stages, EAC has a poor prognosis, with a 5-year survival rate of < 20%.^3^,^9^ However, when early detection and management are possible, the outcome improves significantly.^10^ At present, dysplasia and cancer surveillance in BE follows the Seattle protocol with random 4-quadrant biopsies every 2 cm, which is expensive, time-consuming, and has a sensitivity ranging from 28% to 85% for the detection of HGD/EAC.^11^ When in the hands of expert endoscopists, advanced endoscopic imaging techniques as narrow-band imaging (NBI) and confocal laser endomicroscopy (CLE) can meet optical diagnosis performance thresholds required by the Preservation and Incorporation of Valuable Endoscopic Innovations (PIVI) initiative by the American Society for Gastrointestinal Endoscopy^12^,^13^ [ie, per-patient sensitivity of 90% or greater, a negative predictive value (NPV)] of 98% or greater, and a specificity of at least 80% for detecting HGD or EAC).^11^ However, improving the diagnostic performance of EGDS in the detection of EC regardless of individual expertise is highly desirable, and AI is demonstrating enormous potential in this matter (Table 1).

**TABLE 1:** AI in the Diagnosis of Esophageal Adenocarcinoma

					Performance		
References	AI Model	Study Type	Aim	Endoscopic Technique	Accuracy	Sensitivity	Specificity
de Groof et al^14^	ML—SVM	Retrospective	Detection of early Barrett’s neoplasia	WLE	92%	95%	85%
de Groof et al^15^	DL	Prospective	Detection of early Barrett’s neoplasia	WLE	90%	91%	89%
Ebigbo et al^16^	DL	Prospective	Detection of early Barrett’s neoplasia	WLE	89.9%	83.7%	100%
de Groof et al^17^	DL	Retrospective	AI vs. endoscopists in detection of early Barrett’s neoplasia	WLE AI EE	88% 73%	93% 72%	83% 74%
Swager et al^18^	ML—SVM	Retrospective	AI vs. endoscopists in detection of early Barrett’s neoplasia	VLE AI EE	AUC=0.95 AUC=0.81	90% 85%	93% 68%
van der Sommen et al^19^	ML—SVM	Retrospective	AI vs. endoscopists in detection of early Barrett’s neoplasia	WLE AI Best endoscopist	— —	86% 90%	87% 91%
Ebigbo et al^20^	DL	Retrospective	AI vs. endoscopists in predicting invasion in Barrett’s cancer	WLE AI EE	77% 63%	64% 78%	71% 70%

AI indicates artificial intelligence; AUC, area under the curve; DL, deep learning; EE, expert endoscopists; ML, machine learning; SVM, support vector machine; VLE, volumetric laser endomicroscopy; WLE: white-light endoscopy.

A recent meta-analysis^21^ revealed that AI systems detected BE-related EC with white-light endoscopy (WLE)^14^,^15^,^17^,^19^ or volumetric laser endomicroscopy (VLE)^18^ with a pooled sensitivity of 88% [95% confidence interval (CI), 82.0%-92.1%], pooled specificity of 90.4% (95% CI, 85.6%-94.5%), and an area under the curve (AUC) of 0.96 (95% CI, 0.93-0.99). When compared with general endoscopists operating with standard WLE^17^,^19^ or VLE,^18^ AI performed better than physicians on the detection of neoplastic lesions in BE.^21^ Specifically, AI systems had an AUC of 0.96 (95% CI, 0.94-9.97) versus 0.82, *P*<0.001; sensitivity 90.7% (95% CI, 89.8%-91.5%) versus 72.3% (95% CI, 70.2%-74.3%), *P*<0.001; and specificity 88.0% (95% CI, 87.1%-88.9%) versus 74.0% (95% CI, 72.2%-75.7%), *P*<0.001. However, AI was tested on optimal endoscopic images but not during live EGDS. The retrospective study of Van Riel et al^22^ showed consistent results on still endoscopic images. The AI system detected early BE neoplasms from the public MICCAI 2015 image dataset with AUC of 0.92. Another study applied image data augmentation through Generative Adversarial Networks (GANs) to increase the identification of BE and EAC compared with standard endoscopic images.^23^ The combination of CNNs and GANs allowed to achieve 85% accuracy in the task. Liu et al^24^ recently tested a DL-SVM combined CAD tool to automate the classification of esophageal findings on WLE images. The proposed network achieved accuracy for the classification of cancer, premalignant lesion, and normal esophagus of 77.14%, 82.5%, and 94.23%, respectively. Iwagami et al^25^ trained a DL model to recognize esophageal junctional cancers under WLE and compared its performance with that of expert physicians. The AI system showed a favorable sensitivity of 94%, and specificity of 42% for noncancerous lesions (esophagitis, polyp), which was comparable to that of expert endoscopists (43%). Another DL algorithm was trained to differentiate EC from BE, inflammation, and normal mucosa.^26^ The CAD tool classified the above lesions with overall accuracy of 96%.

AI has shown good performance also in lesion characterization. A pilot study demonstrated that an AI-based system performed as good as international expert physicians in the prediction of submucosal invasion (ie, differentiating stage T1a vs. T1b) in endoscopic images of Barrett’s cancer, having a sensitivity of 77%, a specificity of 64% and an accuracy of 71%.^20^

To enable the integration of CAD systems into clinical practice, research is now concentrating on the real-time use of CAD tools that instantly provide feedbacks to the endoscopist. A preceding to this is the application of AI diagnosis to video clips. Accordingly, in a recent study, a DL model was trained on images and tested on NBI zoom video clips of EAC and NDBE.^27^ The CAD system showed good performance with 83% accuracy, 85% sensitivity, and 83% specificity. Further, de Groof et al^15^ assessed the accuracy of a CAD system for the detection of Barrett’s neoplasia within endoscopic images systematically taken every 2 cm in Barrett’s areas during live endoscopic procedures. The system was tested in real-time on 10 patients with NDBE and 10 patients with BE-related EAC. Standard WLE images were obtained and analyzed live by a DL algorithm that met PIVI threshold with an accuracy of 90%, a sensitivity of 91%, and a specificity of 89%. A different real-time approach was designed by Ebigbo et al.^16^ In their AI system, endoscopic images were randomly captured from the camera livestream during endoscopic procedures. The immediate AI analysis differentiated between NDBE and EAC with a sensitivity of 83.7%, a specificity of 100%, and an accuracy of 89.9%.

### AI in Squamous Dysplasia and Early Esophageal Squamous Cell Carcinoma (ESCC)

ESCC accounts for up to 90% of ECs in lower income countries.^9^ It has a poor prognosis with an overall 5-year survival rate of 18%, which decreases to < 5% when distant metastases are present at diagnosis.^28^ Early detection may potentially improve the outcome of the disease. Endoscopic recognition of early ESCC is challenging, as lesions often pass unrecognized with standard WLE. Lugol’s dye spray chromoendoscopy, virtual chromoendoscopy with NBI and blue-laser imaging (BLI) bright have shown accuracy in the detection of ESCC,^29^–^31^ although nonexpert endoscopists may not perform as good as experts,^31^ limiting their applicability.

To fill the gap, AI has been explored (Table 2). Regarding the diagnosis by NBI or BLI-bright, the performance of AI was compared with that of endoscopy specialists of the Japan Gastroenterological Endoscopy Society.^35^ The sensitivity of the AI system was greater than that of experienced physicians (100% vs. 92%), and the specificity for noncancerous lesions was not significantly lower (63% vs. 69%). DL-based CAD tools have also been challenged to recognize early ESCC under WLE, proving higher accuracy than nonexpert endoscopists and comparable accuracy to expert endoscopists (97.6% vs. 88.8%, and 77.2%, respectively).^32^ Wang et al^41^ developed a single shot multibox detector with a CNN algorithm that performed well in the detection of ESCC using WLE and NBI. The system diagnosed ESCC with 90.9% accuracy. A meta-analysis confirmed that the accuracy of AI in the detection of ESCC was significantly higher when images were analyzed with NBI^33^,^35^,^42^ than WLE,^33^,^42^ being the AUC 0.92 (95% CI, 0.86-1.00) versus 0.83 (95% CI, 0.82-0.84).^21^ When pooled together, the studies that used AI with NBI, WLE, endocytoscopy,^34^ or optical magnifying endoscopy (ME)^35^ to recognize ESCCs, had an AUC of 0.88 (95% CI, 0.82-0.96), a specificity of 92.5% (95% CI, 66.8%-99.5%), and a sensitivity of 75.6% (95% CI, 48.3%-92.5%).^21^

**TABLE 2:** AI in the Diagnosis of ESCC

					Performance		
References	AI Model	Study Type	Aim	Endoscopic Technique	Accuracy	Sensitivity	Specificity
Cai et al^32^	DL	Retrospective	Detection of ESCC	WLE	91.4%	98%	85%
Guo et al^33^	DL	Retrospective	Detection of ESCC	NBI images NBI videos	AUC=0.989 100%	98% 100%	95% 100%
Kumagai et al^34^	DL	Prospective	Detection of ESCC	ECS	AUC=0.85	39%	98%
Ohmori et al^35^	DL	Retrospective	Detection of ESCC	WLE NBI/BLI ME—BLI/NBI	81% 77% 77%	90% 100% 98%	76% 63% 56%
Tokai et al^36^	DL	Retrospective	Detection of ESCC	WLE/NBI	96%	—	—
			Estimating invasion depth of ESCC	WLE	SM1=93% SM2=97%	— —	— —
				NBI	SM1=97% SM2=100%	— —	— —
Nakagawa et al^37^	DL	Retrospective	Estimating invasion depth of ESCC	WLE/NBI/BLI	SM1=93% SM2=90%	95% 94%	79% 75%
				ME—WLE/NBI/BLI	SM1=90 SM2=92	92% 94%	79% 86%
Shimamoto et al^38^	DL	Retrospective	Estimating invasion depth of ESCC	WLE/NBI/BLI ME—WLE/NBI/BLI	87% 89%	50% 71%	99% 95%
Everson et al^39^	DL	Retrospective	Detection of abnormal IPCL	ME-NBI	98%	99%	97%
Zhao et al^40^	DL	Retrospective	Classification of IPCL	ME-NBI	89%	—	—

AI indicates artificial intelligence; AUC, area under the curve; BLI, blue-laser imaging; DL, deep learning; EAC, esophageal adenocarcinoma; ECS, endocytoscopic system; ESCC, esophageal squamous cell carcinoma; IPCL, interpapillary capillary loop; ME, magnified endoscopy; NBI, narrow-band imaging; SM, submucosal; WLE, white-light endoscopy.

AI has also been tested on the characterization of mucosal invasion of ESCC through the analysis of esophageal intrapapillary capillary loops (IPCLs), which are microvascular structures on the surface of the esophagus. IPCLs appear as brown loops on ME with NBI and show morphologic changes that strictly correlate with neoplastic invasion depth of ESCC, allowing intraprocedural decisions for endoscopic resections.^43^,^44^ However, optical classification of IPCL requires experience and is mastered by experts only. Accordingly, it was developed an AI-based automated IPCL classification whose accuracy was significantly higher than that of endoscopists with <15 years of experience.^40^ A CNN-based AI system^39^ was trained with sequential high-definition ME-NBI images from 17 patients (10 ESCN, 7 normal), and distinguished abnormal IPCL patterns with 93.7% accuracy. The sensitivity and specificity to classify abnormal IPCL patterns were 89.3% and 98%, respectively. In another study, AI estimated the invasion depth of ESCC from NBI/WLE images better than 13 expert endoscopists, showing a sensitivity of 84.1% versus 78.8%, a specificity of 73.3% versus 61.7%, and an accuracy of 80.9% versus 73.5%.^36^ Another AI system showed good performance in differentiating mucosal and submucosal microinvasive (SM1) cancers from the submucosal deep invasive (SM2/3) ones with a sensitivity of 90.1%, a specificity of 95.8%, and an accuracy of 91.0%. The performance of the system was comparable to that of experienced physicians.^37^ AI also showed comparable results to expert clinicians when classifying IPCLs from video clips.^45^ A CNN model showed accuracy, sensitivity, and specificity of 91.7%, 93.7%, and 92.4%, respectively, in the recognition of abnormal IPCLs during the analysis of video frames.

Growing proficiency in AI systems allowed the development of real-time operating CAD tools. A CAD video model^33^ was capable of processing at least 25 frames/s of NBI images in < 100 ms with encouraging performance. The dataset included precancerous lesions, early ESCC, and nonpathologic findings. When analyzing non-ME videos, the per-frame and per-lesion sensitivity of the AI system were 60.8% and 100%, respectively. Notably, the per-frame sensitivity increased to 96.1%, and the per-lesion sensitivity remained stable to 100% with ME videos. Another recent study^46^ compared the ability of a CAD system to that of 13 expert endoscopists to identify and characterize suspicious lesions from video clips of NBI esophagoscopies. Regarding detection performance, AI sensitivity was significantly higher than that of experts, being 91% versus 79%, whereas AI specificity and accuracy were lower, being 51% versus 72%, and 63% versus 75%, respectively. As for differentiating cancerous from noncancerous lesions, the AUC showed that the AI system had significantly better diagnostic performance than physicians, being the sensitivity 86% versus 74%, the specificity 89% versus 76%, and the accuracy 88% versus 75%. Yang et al^47^ developed a real-time operating AI system that could detect 100% and 95% of early ESCC from ME and non-ME WLE video clips, respectively. Waki et al^48^ challenged the AI to diagnose ESCC from video clips simulating a situation in which the CAD tool could assist clinicians during routine EGD. The assistance of AI significantly improved the sensitivity for ESCC diagnosis by 2.7%. Similarly, Li et al^49^ compared AI-aided detection of ESCC under WLE and NBI, and evaluated the yield of its support to endoscopists. The accuracy of the AI system with NBI and WLE was 94.3% and 89.5%, respectively, whereas the average accuracy of endoscopists was 81.9%. Remarkably, the assistance of AI allowed endoscopists to achieve the highest accuracy of 94.9% with NBI and WLE.

A recently developed CAD system^38^ achieved a favorable performance at estimating the invasion depth of ESCC in video images with nonmagnifying WLE or ME with NBI/BLI. Accuracy, sensitivity, and specificity of the AI system with non-ME versus those of endoscopists with up to 15 years of experience, were 87% versus 85%, 50% versus 45%, and 99% versus 97%. Good performance was also achieved when comparing the AI system with ME, where accuracy, sensitivity, and specificity were 89% versus 84%, 71% versus 42%, and 95% versus 97%, respectively. AI could potentially help with other endoscopic techniques that require much experience. Accordingly, a clinical trial aiming to evaluate the automatic diagnosis of early ESCC with probe-based CLE is currently recruiting (NCT04136236). The primary outcome will be to test the real-time diagnostic performance of the AI system with probe-based CLE and the secondary to compare AI performance to that of endoscopists.

### AI in Benign Esophageal Diseases: Gastroesophageal Reflux, Motility Disorders, Esophagitis, and Varices

The huge computing power of AI facilitates and optimizes the analysis of large amounts of data at once, allowing to recognize complex nonlinear interactions between variables. Accordingly, AI has been applied to esophageal benign disorders (Table 3) including GERD, primary motility disorders, EoE, cytomegalovirus (CMV) and herpes simplex virus (HSV) esophagitis, and esophageal varices.

**TABLE 3:** AI in the Diagnosis of Benign Esophageal Diseases

					Performance		
References	AI Model	Study Type	Aim	Diagnostic Tool	Accuracy	Sensitivity	Specificity
Pace et al^50^	ML	Prospective	Distinction between GERD and non-GERD based on symptoms	Questionnaire	100%	—	—
Horowitz et al^51^	Data mining	Prospective	Distinction between GERD and non-GERD based on symptoms	Questionnaire	AUC=0.78	70%-75%	63%-78%
Pace et al^52^	ML	Prospective	Distinction between NERD and EE	Questionnaire	NERD 62.2% EE 70.9%	—	—
Rogers et al^53^	Decision tree analysis	Prospective	Automate extraction of pH-impedance metrics	pH-impedance tracings	88.5%	—	—
Rogers et al^53^	Decision tree analysis	Prospective	Predict response to GERD management	pH-impedance tracings	AUC=0.77	—	—
Gulati et al^54^	DL	Prospective	Endoscopic diagnosis of GERD	NF-NBI	AUC=0.83	67%	92%
Sallis et al^55^	ML	Prospective	Diagnosis of EoE based on mRNA transcripts from esophageal biopsies	Esophageal biopsies	AUC=0.98	91%	93%
Santos et al^56^	Multilayered back-propagation ANN	Prospective	Diagnosis of esophageal motility pattern	Stationary esophageal manometry tracings	82%	—	—
Lee et al^57^	DL	Retrospective	AI vs. endoscopist in differential diagnosis HSV vs. CMV esophagitis	WLE AI Endoscopists	100% 52.7%	100% —	100% —

AI indicates artificial intelligence; ANN, artificial neural network; AUC, area under the curve; CMV, cytomegalovirus; DL, deep learning; EE, erosive esophagitis; EoE, eosinophilic esophagitis; GERD, gastroesophageal reflux disease; HSV, herpes simplex virus; ML, machine learning; NERD, nonerosive reflux disease; NF-NBI, near-focus narrow-band imaging; WLE, white-light endoscopy.

GERD is defined as the presence of troublesome symptoms caused by gastroesophageal reflux and/or esophageal mucosal lesions.^58^ It has a spectrum of symptoms (eg, heartburn, regurgitation, noncardiac chest pain) that overlap with reflux hypersensitivity and functional heartburn,^59^–^62^ which can make clinical distinction difficult.

In 2005, an AI system based on ML was developed to discriminate, based on symptoms solely, between patients with a pathologic esophageal acid pH exposure with or without esophagitis, and normal individuals.^50^ Patients were asked to fill in the Gastro-Esophageal Reflux Questionnaire (GERQ) proposed by the Mayo Clinic,^63^ and underwent an EGD and/or a 24-hour esophageal pH-metry. Among patients, 103 had an objectively confirmed GERD, and 56 were normal. The AI system automatically selected the 45 most relevant variables from the GERQ (collectively referred to as QUID, “*QUestionario Italiano Diagnostico*”) (Table 4), and this allowed the CAD tool to reach a predictive accuracy up to 100%, as it correctly predicted GERD in all 103 patients. Another study with ML combined AI and the QUID questionnaire to differentiate between GERD patients with and without erosive esophagitis (ie, nonerosive reflux disease).^52^ The CAD tool successfully distinguished between GERD and normal patients but failed to discriminate erosive esophagitis from nonerosive reflux disease based solely on symptoms evaluation. Horowitz et al^51^ developed and validated a shorter questionnaire of 15 variables (Table 5) aiming to discriminate, through an ANN-based algorithm, GERD from non-GERD patients. The sensitivity of the model was 70% to 75%, the specificity 63% to 78%, and the AUC 0.787.

**TABLE 4:** Variables Included in the *QUestionario Italiano Diagnostico* Questionnaire^52^

Heartburn	Persistent Gastric/Intestinal Pain	Prescribed Examinations for GERD	Relative With Gastroduodenal Disorder	Medical Visits for GERD
Hiatus hernia	Cough intensity	Aspirin, frequency of use	Marital status	Episodes of breathlessness
Frequency of chest pain	Periodic frequency of swallowing problems	Antirheumatic drugs, frequency of use	Cough frequency/year	Asthma
Waking at night–retrosternal pain	Intensity of swallowing problems	esophageal dilation	Slow walk—chest pain	Belching
Pneumonia	Frequency of medical check-ups	Variation in weight in past year	Heart therapy	Sibilant rhonchi or wheezing
Cough	Heart disorders	Coffee drinker	Intensity of chest pain	Cough at night
Retrosternal pain	Vomiting (frequency)	Regular smoker	Chest pain in past year	Ingestion of beverages—chest pain
Interference with daily activities	Acid reflux in mouth	Lump in throat	Alcohol units/week in past year	Total acid reflux
Sleeping semisupine	Esophageal surgery	Health state during past year	Hiccups	Intensity of acid reflux

GERD indicates gastroesophageal reflux disease.

**TABLE 5:** Symptoms/Signs Evaluated in the Artificial Neural Network-based Questionnaire Proposed by Horowitz et al^51^ to Primary Care Physicians to Diagnose Gastroesophageal Reflux Disease

Abdominal Pain	Belching	Halitosis
Chest pain	Heartburn	Relief with antacid medications
Bloating	Regurgitation	Stress
Nausea	Dysphagia	Bend/lie aggravation
Vomiting	Sour taste	Heavy meal aggravation

Moving from categorical to numerical data, AI has also been applied to pH-impedance studies. Twenty-four-hour pH-impedance monitoring is used to quantify reflux episodes and acid exposure time (AET) to rule in or out the diagnosis of GERD.^64^–^68^ The mean nocturnal baseline impedance is a novel metric that demonstrated to increase GERD diagnostic yield and predict treatment outcome.^65^,^69^–^74^ A recently published proof-of-concept study showed that AI has the potential to automate the extraction and to elaborate useful novel AI pH-impedance metrics. CNN was considered inadequate to be applied to impedance tracings, and a python-based decision tree analysis algorithm was developed to analyze raw pH-impedance data.^53^ The AI system autonomously evaluated 2049 pH-impedance events with an accuracy of 88.5%, and calculated recumbent and upright values of baseline impedance (AIBI). The upright AI divided by recumbent AI ratio (U:R AIBI ratio) segregated responders to treatment from controls and nonresponders regardless of treatment status upon pH-impedance recording. Moreover, the U:R AIBI ratio at 5 cm above the lower esophageal sphincter outperformed total AET in predicting response to medical therapy in those with AET >6% (AUC: 0.766 vs. 0.606, respectively).

In a recent clinical trial (NCT04268719), an image-driven AI model for the diagnosis of GERD was developed. A published abstract demonstrated the potential of AI-driven near-focus NBI endoscopy for the real-time diagnosis of GERD through the recognition of regions of interests and IPCLs.^54^ The CNN-based model could diagnose GERD in real-time during endoscopic procedures with a sensitivity, specificity, and AUC up to 67%, 92%, and 0.83, respectively.

The feasibility of applying ANNs in the recognition and classification of primary esophageal motor disorders has also been investigated.^56^ Two different ANN models were trained to recognize normal and abnormal swallow sequences of pressure wave patterns of conventional stationary manometry recordings. The model correctly classified >80% of swallow sequences, diagnosing 100% of cases of achalasia, 100%, of nutcracker esophagus, 80% of ineffective esophageal motility, 60% of diffuse esophageal spasm, and 80% of normal motility. However, the study took place in 2006, when high-resolution manometry recordings and current highly sophisticated AI algorithms were not available.

Early encouraging applications of AI to EoE have also been reported. EoE is a chronic, local, progressive, T-helper type 2 immune-mediated esophageal disorder.^75^–^78^ Clinical manifestations vary according to the age of diagnosis, and a timely diagnosis may be difficult.^77^,^79^ Accordingly, an AI-based automated algorithm was developed to assist in the diagnosis of EoE.^55^ The AI system elaborated a diagnostic probability score for eosinophilic esophagitis (pEoE) based on esophageal mRNA transcripts from biopsies of EoE patients, including genes encoded by the EoE transcriptome.^76^ During the process, individual transcripts were automatically assigned weights by the system. Interestingly, established EoE markers (eg, eotaxin and periostin)^76^ were weighed higher. For validation, the pEoE score was applied to a set of external patients in a blinded fashion. A pEoE score ≥25 detected EoE patients with a sensitivity of 91%, a specificity of 93%, and AUC 0.985. Importantly, the pEoE score improved the diagnosis of equivocal EoE cases with 84.6% accuracy, distinguishing EoE from GERD. In treatment-responsive patients (ie, < 5 eosinophils/HPF), the pEoE score decreased below the diagnostic cutoff of 25 and remained ≥25 in the 1 patient whose eosinophilia did not resolve completely (5 <eosinophils/HPF<15). To date, no studies or clinical trials combining AI and endoscopic evaluation of EoE have been published.

AI has shown to be useful also in the optical endoscopic differential diagnosis of CMV versus HSV esophagitis. CMV and HSV esophagitis have overlapping endoscopic findings and are infrequent in clinical practice, which makes the diagnosis challenging and hampers the start of a targeted treatment before the histopathologic diagnosis. Accordingly, Lee et al^57^ trained a DL system to differentiate CMV from HSV esophageal ulcers with impressive results. The accuracy, sensitivity, and specificity of the CAD tool were 100%, largely outperforming endoscopists, whose accuracy was only 52.7%.

Finally, Guo et al^80^ trained a DL system to classify multiple GI lesions. Among these, esophageal varices could be automatically diagnosed by the system with 90.5% sensitivity.

## AI IN GASTRIC DISEASES

### AI in Gastric Cancer (GC) and Chronic Atrophic Gastritis (CAG)

GC is one of the 5 most common cancer-related diagnoses globally and the third leading cause of death in cancer patients. Early recognition of premalignant lesions is the main goal to reduce the burden of the disease. Endoscopic evaluation is the standard of care in assessing the gastric mucosa. A recent systematic review and meta-analysis evaluated the data regarding AI and GC demonstrating the expanding role and the favorable impact of this technology for current and upcoming years.^81^ Hirasawa et al^82^ developed the first AI system for the detection of GC using DL. Although demonstrating a high sensitivity (92.2% for lesions of 5 mm or less; 98.6% in lesions of 6 mm or more), its positive predictive value (PPV) was 30.6%, due to misdiagnosis of mild-moderate CAG and intestinal metaplasia lesions. In this regard, another study showed that a DL approach had a significantly higher accuracy than experts in the assessment of CAG.^83^ Other AI systems have been developed since (Table 6), such as the one proposed by Horiuchi et al^89^ which differentiated GC from gastritis with 85.3% accuracy, 95.4% sensitivity, and 71.0% specificity. Wu et al,^85^ tested a DL system in the detection of GC. The sensitivity, specificity, accuracy, PPV, and NPV were 94.0%, 91.0%, 92.5%, 91.3%, and 93.8%, respectively. Li et al^84^ developed an AI model based on CNN to differentiate between noncancerous and early gastric mucosal lesions under ME. The proposed system showed a sensitivity, specificity, and accuracy of 91.1%, 90.6%, and 90.9%, respectively, in the diagnosis of early GC, which were significantly higher than those of nonexpert endoscopists. Lee et al^88^ combined a residual network with transfer learning to distinguish between GC, ulcers, and normal gastric mucosa with almost 90% accuracy. Zhang et al^87^ used the CNN DenseNet to identify CAG lesions, with diagnostic accuracy, sensitivity, and specificity of 0.94, 0.95, and 0.94. The classification accuracy between mild, moderate, and severe CAG was 0.93, 0.95, and 0.99 demonstrating higher detection rates for moderate and severe cases. Ueyama et al^86^ tested a DL system on 2300 ME-NBI images (1430 GC images), achieving 98.7% accuracy, 98% sensitivity, and 100% specificity for the diagnosis of GC. In a multicenter study,^92^ the performance of AI with ME-NBI was similar to that of senior endoscopists and better than that of junior endoscopists. Interestingly, the diagnostic ability of endoscopists improved significantly after referring to the results provided by the AI system, providing insights into a useful application of AI in routine practice.

**TABLE 6:** AI in the Diagnosis of Gastric Cancer and CAG

					Performance		
References	AI Model	Study type	Aim	Diagnostic Tool	Accuracy	Sensitivity	Specificity
Li et al^84^	DL	Retrospective	Diagnosis of GC	ME-NBI	90.9%	91.1%	90.6%
Hirasawa et al^82^	DL	Retrospective	Diagnosis of GC	WLE, NBI, IC	—	92.2%-98.6%	—
Wu et al^85^	DL	Retrospective	Diagnosis of GC	WLE, NBI, BLI	92.5%	94.0%	91.0%
Ueyama et al^86^	DL	Retrospective	Diagnosis of GC	ME-NBI	98.7%	98.0%	100%
Zhang et al^87^	DL	Retrospective	Detection of CAG	WLE	94.0%	95.0%	94.0%
Lee et al^88^	DL	Retrospective	Differential diagnosis GC vs. gastric ulcer	WLE	77.1%-90%	—	—
Horiuchi et al^89^	DL	Retrospective	Differential diagnosis GC vs. gastritis	ME-NBI	85.3%	95.4%	71.0%
Zhu et al^90^	DL	Retrospective	Characterization of GC invasion depth	WLE	89.1%	76.5%	95.5%
Nagao et al^91^	DL	Retrospective	Characterization of GC invasion depth	WLE, NBI, IC	94.5%	84.4	99.4%

AI indicates artificial intelligence; BLI, blue-laser imaging; CAG, chronic atrophic gastritis; DL, deep learning; GC, gastric cancer; IC, indigo-carmine dye contrast; ME, magnified endoscopy; NBI, narrow-bad imaging; WLE, white-light endoscopy.

AI has also been applied to the recognition of GC invasion depth. Importantly, patients with cancers extending within the mucosa or submucosal layer could benefit from curative endoscopic resection regardless of lymph node involvement. However, the prediction of invasion depth is challenging in clinical practice. Accordingly, a CNN detection system was developed to determine GC invasion depth based on WLE images.^90^ The model reached an overall accuracy of 89.1%, sensitivity of 76.5%, and specificity of 95.5%. Of note, the system had a significantly higher accuracy of 17.2% and a higher specificity of 32.2% than expert endoscopists. Similarly, Nagao et al^91^ compared AI with WLE, NBI, and indigo-carmine dye contrast imaging in characterizing GC invasion depth. The authors calculated accuracies of 94.5%, 94.2%, and 95.5%, respectively, without significant differences among imaging techniques.

### AI in the Detection of *Helicobacter pylori* (Hp) Infection

Hp is a gram-negative bacterial pathogen that selectively colonizes the gastric epithelium.^93^ The infection is a leading cause of gastroduodenal pathology.^94^ In 1994, the International Agency for Research on Cancer labeled Hp as a definite (group I) carcinogen for GC.^95^ Indeed, the infection is the major cause of chronic gastritis that sequentially causes precancerous modifications, namely atrophic gastritis, intestinal metaplasia, dysplasia and ultimately, GC. Accordingly, the eradication of Hp has the potential to prevent the development of preneoplastic lesions of gastric mucosa.^96^ More than 50% of the world’s population is infected with Hp,^97^ which makes the widest possible diagnosis and eradication of the infection a matter of global health interest. No single endoscopic approach provides validated accuracy for optical diagnosis of Hp infection at present, and endoscopic biopsies are required.^7^ Recently, several AI models have shown potential to overcome biopsy sampling, or provide “heat maps” for targeted biopsies, and allow accurate optical detection of Hp-infected gastric mucosa to achieve a prompter diagnosis (Table 7).

**TABLE 7:** AI in the Diagnosis of *Helicobacter pylori* Infection

					Performance		
References	AI Model	Study Type	Aim	Diagnostic Tool	Accuracy	Sensitivity	Specificity
Shichijo et al^98^	DL	Retrospective	Detection Hp infection	WLE	87.7%	88.9%	87.4%
Yasuda et al^99^	DL	Retrospective	Detection Hp infection	LCI	87.6%	90.5%	85.7%
Zheng et al^100^	DL	Retrospective	Detection Hp infection	WLE Per-image analysis Per-patient analysis	84.5% 93.8%	81.4% 91.6%	90.1% 98.6%
Shichijo et al^101^	DL	Prospective	Detection of Hp-eradicated gastric mucosa	WLE	84%	—	—
Nakashima et al^102^	DL	Prospective	Detection Hp infection	WLE BLI-bright LCI	AUC=0.66 AUC=0.96 AUC=0.95	66.7% 96.7% 96.7%	60% 86.7% 83.3%
Itoh et al^103^	DL	Prospective	Detection Hp infection	WLE	0.956	86.7%	86.7%
Huang et al^104^	RFSNN	Prospective	Detection Hp infection	WLE	—	85.4%	90.9%
Huang et al^105^	SVM	Retrospective	Detection Hp infection	WLE	86%	—	—

AI indicates artificial intelligence; AUC, area under the curve; BLI, blue-laser imaging; DL; deep learning; Hp, *Helicobacter pylori;* LCI, linked color imaging; RFSNN, refined feature selection with neural network; SVM, support vector machine; WLE, white-light endoscopy.

An early study by Huang et al^104^ investigated a refined feature selection with neural network model for prediction of Hp-related gastric histologic features. The authors trained the AI system with endoscopic images of 30 prospectively enrolled dyspeptic patients with and without histologically confirmed Hp infection, and then tested the performance of the model on endoscopic images on 74 dyspeptic patients previously unknown to the system. The refined feature selection with neural network model showed a sensitivity of 85.4% and a specificity of 90.9% for the detection of Hp infection, and an accuracy >80% in predicting the presence of gastric atrophy, intestinal metaplasia, and the severity of gastric inflammation. Another study^105^ aimed to automate the recognition of histologic parameters proposed by the updated Sydney system.^106^ The authors applied a SVM-based AI model to WLE images, which achieved an accuracy rate of 86% for the detection of Hp by image analysis for any topographic locations over the gastric antrum, body, or cardia. More recently, several CNN models have been engineered to support the optical diagnosis of Hp. In 2017, Shichijo et al^98^ developed 2 CNN-based CAD tools. The first was trained with WLE images classified according to the presence or absence of Hp infection. The system achieved an accuracy of 83.1%, a sensitivity of 81.9%, and a specificity of 83.4% in 198 seconds for the detection of Hp infection. The second CNN system was trained with images classified according to anatomic locations, achieving an accuracy of 87.7%, a sensitivity of 88.9%, and a specificity of 87.4% in 194 seconds for the diagnosis of Hp infection. Notably, the diagnostic performance of the second CNN model was significantly higher than that of 23 endoscopists, who achieved an accuracy of 82.4% in the considerably longer time of 230 minutes. A subsequent study further challenged a CNN model to recognize the gastric mucosa of Hp-eradicated patients.^101^ The AI system showed 84% accuracy for the detection of Hp-eradicated gastric mucosa on WLE images. Itoh et al^103^ trained a CNN-based CAD system with WLE images of Hp-positive and negative patients. The model performed well for detection of Hp infection on prospectively collected endoscopic images, with an AUC of 0.956, a sensitivity and a specificity of 86.7%. Similarly, another study trained and validated a DL model with 2 different retrospectively collected WLE sets of images from a total of 1959 patients.^100^ The system showed high accuracy, with a per-image AUC of 0.93, sensitivity of 81.4%, specificity of 90.1%, and accuracy of 84.5%. Per-patient AUC was 0.97, with sensitivity 91.6%, specificity 98.6%, and accuracy 93.8%. Recently, AI has been applied to advanced endoscopic imaging for the diagnosis of Hp infection, including linked color imaging (LCI) and BLI-bright. LCI is a new image-enhanced endoscopy system developed by Fujifilm Co. (Tokyo, Japan) that enhances even slight mucosal color differences. The technique has already proven accuracy in the optical diagnosis of active Hp infection.^99^,^107^ Yasuda et al^99^ developed an SVM-based classification algorithm to detect Hp infection on LCI endoscopic images. The authors trained an AI system by SVM, using LCI images of infected and uninfected patients, and then compared the performance of the CAD system to that of an expert in LCI, a gastroenterologist specialist, and a senior resident. For the diagnosis of Hp infection, the AI system proved accuracy of 87.6%, sensitivity of 90.5%, and specificity of 85.7%. Of note, the system significantly outperformed the senior resident. The performance of the LCI expert and the gastroenterology specialist were comparable to that of AI system, with nonsignificant differences. Nakashima et al^102^ assessed the performance of a CNN-based AI system for the diagnosis of Hp infection comparing WLE and image-enhanced endoscopy. Three still images of the lesser curvature of the stomach were taken with WLE, BLI-bright, and LCI for each of the 222 included patients (105 infected). The images of 162 patients were used to train the AI system, those of 60 patients were used for validation. BLI-bright and LCI significantly outperformed WLE in a comparable amount of time. In detail, the AUC for BLI-bright and LCI were 0.96 and 0.95, whereas the AUC for WLE was 0.66. BLI-bright had a sensitivity of 96.7% and a specificity of 86.7%, LCI had a sensitivity of 96.7% and a specificity of 83.3%, and WLE had a sensitivity of 66.7% and a specificity of 60.0%.

## DISCUSSION AND CONCLUSION

AI is attracting increasing attention in diagnostic imaging and complex medical data analysis. It has been observed that CAD tools provide an interpretable universal method for clinical instrumental and endoscopic diagnosis of GI diseases (Table 8), virtually eliminating interobserver variability and reducing the rate of blind spots during EGDS.^108^ Despite the in vivo application of AI is relatively recent, exciting results have already been achieved.

**TABLE 8:** Summary Table: Current Application of AI in the Upper Gastrointestinal Tract

Target Organ	Type of Disease	Disease	Current Application of AI	Future Perspectives of AI
Esophagus	Malignant/premalignant	Barrett’s esophagus	Detection	Anticipate the diagnosis of cancer and improve the prognosis of the disease
		Adenocarcinoma	Detection Characterization (invasion depth)	
		Squamous dysplasia	Detection	
		Interpapillary capillary loops	Detection Characterization (morphology)	
		Squamous cell carcinoma	Detection Characterization (invasion depth)	
	Benign	GERD	Endoscopic automated diagnosis of abnormal acid exposure Noninvasive diagnosis based on symptoms Differential diagnosis NERD vs. ERD Automated extraction of metrics from pH-impedance tracings Prediction of response to treatment	Automate the diagnosis of NERD during endoscopy, provide a noninvasive conclusive diagnosis of GERD, and make quicker the interpretation of pH-impedance tracings, which currently require a time-consuming manual revision
		Esophagitis	Differential diagnosis between CMV and HSV esophagitis	Anticipate diagnosis and start treatment without waiting for the histologic diagnosis
		Dysmotility	Detection Characterization of motility pattern (manometric diagnosis)	Automate and speed-up the diagnosis of motility disorders
		Varices	Detection Diagnosis	Automate the detection and classification of varices during routine endoscopy
Stomach	Malignant/premalignant	Chronic atrophic gastritis	Detection	Anticipate the diagnosis of cancer and improve the prognosis of the disease
		Gastric cancer	Detection Characterization (invasion depth)	
	Benign	*Helicobacter pylori* infection	Detection	Automated diagnosis regardless of confounding factors (eg, PPI therapy), start treatment without waiting for the histologic diagnosis
		Gastric ulcer	Differential diagnosis with cancer	Anticipate the diagnosis and start treatment without waiting for the histologic diagnosis

AI indicates artificial intelligence; CMV, cytomegalovirus; ERD, erosive reflux disease; GERD, gastroesophageal reflux disease; HSV, herpes simplex virus; NERD, nonerosive reflux disease; PPI, proton-pump inhibitors.

A recent meta-analysis confirmed the potential of AI to increase the diagnostic yield and reduce underdiagnosis of neoplastic lesions.^109^ AI systems had a sensitivity of 90%, a specificity of 89%, a PPV of 87%, and an NPV of 91% without significant performance differences in the diagnosis of ESCC, BE-related EAC, or GC. In addition, CAD tools showed good performance in the characterization of submucosal invasion of esophageal and gastric cancerous lesions.^36^–^40^,^43^,^44^,^90^ This has relevant therapeutic and prognostic implications as early lesions are amenable of endoscopic treatment.^110^

AI also proved utility to support both clinical and endoscopic diagnosis of benign upper GI pathology. AI systems helped in the development of questionnaires that accurately predict GERD and differentiate disease from normality.^50^–^52^ AI models autonomously extracted and analyzed pH-impedance tracings and also individuated a novel pH-impedance metric, that is, mean nocturnal baseline impedance, which enables to segregate responders from nonresponders to GERD treatment, thus reducing reporting times and virtually improving reflux management.^53^ The possibility of a real-time endoscopic GERD diagnosis was also shown.^54^

A CAD tool could recognize stationary manometry motor patterns with accuracy,^56^ but the application of novel CAD tools to high-resolution manometry recordings is yet to be evaluated.

The ability of AI to analyze complex variables interactions allowed the development of a score that could predict EoE with good accuracy.^55^ Although NBI can accurately differentiate EoE from control patients,^111^ there are no published studies that combine NBI and AI for the diagnosis of EoE. It is tempting to speculate that AI could support the endoscopic diagnosis of EoE when typical endoscopic findings are absent.^77^

In addition, AI demonstrated impressive utility in the real-time endoscopic diagnosis of infrequent forms of esophagitis (ie, CMV and HSV), which are often misdiagnosed even by experts.^57^

As regards Hp, a recent meta-analysis confirmed that AI algorithms are reliable predictors of infection with AUC of 0.92 (95% CI, 0.90-0.94), pooled sensitivity of 0.87 (95% CI, 0.72-0.94), and specificity of 0.86 (95% CI, 0.77-0.92).^112^

The world of AI sets the groundwork for the medicine of the future, and we should brace ourselves for the integration of CAD tools into clinical practice. However, reliance on AI tools should not replace clinical judgment. This is because AI has a black-box nature and is currently burdened with high costs. In addition, the high computational power of AI algorithms carries the risk of overfitting, in which the model is too tightly fitted to the training data and does not generalize towards new data.^113^ More real-time high-quality studies are needed to expand these early results that, if confirmed, will represent a revolution of routine clinical gastroenterological practice.
